# Silicea Gastrointestinal Gel Improves Gastrointestinal Disorders: A Non-Controlled, Pilot Clinical Study

**DOI:** 10.1155/2012/750750

**Published:** 2012-03-04

**Authors:** B. Uehleke, M. Ortiz, R. Stange

**Affiliations:** ^1^Department for Natural Medicine, Charité-Universitätsmedizin Berlin, Immanuel Hospital, 14109 Berlin, Germany; ^2^Institute of Complementary Medicine, University Hospital Zürich, 8091 Zürich, Switzerland; ^3^University of Health & Sport, 10367 Berlin, Germany; ^4^Institute for Social Medicine, Epidemiology and Health Economics, Charité University Medical Center, 10098 Berlin, Germany

## Abstract

*Aim*. To investigate efficacy and tolerability of Silicea Gastrointestinal Gel in patients with gastrointestinal disorders. *Methods*. Open, prospective pivotal phase IV study with oral Silicea Gastrointestinal Gel over 6 weeks. Symptom score was part 1 of the Nepean Dyspepsia Index: 15 questions addressing intensity, frequency and impact of upper abdominal symptoms. 10 lower abdominal symptoms were asked analogously. A responder showed reduction of score of >50%. *Results*. 62 of 90 patients were evaluated per protocol. Upper and lower abdomen sum scores decreased already in the first three weeks (*P* < 0.001), which continued the following three weeks (*P* < 0.01). Mean symptom score for upper abdomen decreased from 52.2 ± 31.0 to 33.7 ± 28.7 (or by 35.4%; responder rate 37%); for lower from 39.6 ± 24.7 to 22.6 ± 21.7 (by 42.9%; responder rate 46%). Subgroups with diarrhea, IBS and GERD presented highest responder rates. 6% of patients reported adverse reactions with probable or possible relationship to the test product. *Conclusions*. Silicea Gastrointestinal Gel seems suitable beyond infectious acute gastrointestinal disorders. Responses are relevant for chronic functional disorders, but it remains unclear, how much of that might be placebo-effect. Controlled studies are recommended in gastrointestinal syndromes like IBS or GERD.

## 1. Introduction

About 20–30% of people in the western world experience chronic gastrointestinal complaints relating to functional bowel disorders [1], half of those seeking medical attention [2, 3]. 

The spectrum of symptoms such as pain, nausea, emesis, distension, flatulence, and diarrhea is linked to various conditions, from acute infections to chronic dysfunctions. After exclusion of a somatic disorder, such chronic symptoms are usually categorized as either nonulcer dyspepsia (NUD) or irritable bowel syndrome (IBS). Comorbidity, respectively, consecutive appearance of both entities is frequent. However, the diagnosis can be challenging particularly because of variability and overlap of symptoms [4]. Functional gastrointestinal complaints are often treated with drugs such as proton pump inhibitors (PPIs) or prokinetics. The overlap of upper and lower GI symptoms might explain why PPIs do not provide complete symptom relief in patients with GERD [5] (gastroesophageal reflux disease) and corresponding improvement in health-related quality of life [6]. But PPIs might also induce gastrointestinal symptoms, especially after stopping therapy [7].

10–15% of the western world is affected by IBS [8, 9] and about 5% seeks medical attention. IBS is associated with high direct and productivity costs. The prevalence of simple constipation is also as high as 15% in North America [10].

Interpreting efficacy studies in functional dyspepsia is difficult since a variety of test instruments are used, many of which have not been validated. Additionally, most patients continue to self-treat with over-the-counter medication. Some herbal products have been shown to be helpful in treating gastrointestinal problems [11], and some of these studies have been conducted in comparison with standard prokinetic drugs [12]. For many other over-the-counter medications, research about use, benefit, and risk is lacking. Evidence of efficacy of IBS drug therapies with bulking agents, antispasmodics, and antidepressants is weak, particularly because of the problem of valid outcome measures [13].

Silicea Gastrointestinal Gel is classified as a Class IIa medical device in Germany due to the fact that it is not absorbed during digestion. 100 mL of the silicon gel contain 3.5 g silicon dioxide (silica). The silicon dioxide is mixed with water to form a gel. Molecules (acids, ions, organic poisons, gas, and bacteria) are adsorbed to this structure. Silicic acid is also extremely hygroscopic; therefore also water molecules bind to the primary hydrophilic adsorption sites. This enables silica gel to have very large surface areas. For example, a surface area of 300 m^2^/g has been measured, with a particle size of 3.5 to 4.5 *μ*m. A structure with secondary channels as adsorption cavities has been described as a treatment for infectious diarrhea. Relevant binding capacity was found for staphylococcal enterotoxin C1 and bacteria (*E. coli*), which was comparable to that of activated carbon, and was not affected by acidic pH [14]. Taking into consideration the negative charge of the silicon dioxide particles and predominantly negative charge of the bacteria surfaces, the high binding capacity for bacteria may be explained by flocculating aggregation via bridge-building anorganic cations. The silica gel structure binds water and is not affected in the upper intestinal tract. Furthermore silicic acid could provide local protection to the gastrointestinal mucous membrane. Clinically, decrease in duration of diarrhea, stool frequency [15], abdominal pain, distension, nausea, and vomiting has been described [16]. It is currently unknown, however, which gastrointestinal symptoms respond best to silica gel and whether acute or chronic symptoms may be influenced by this treatment.

## 2. Materials and Methods

### 2.1. Ethics

This work has been carried out in accordance with the Declaration of Helsinki (2000) of the World Medical Association. Since this was an observational study of an over-the-counter product, ethics committee approval was not necessary. Patients signed informed consent to electronic processing of their pseudonymized data.

### 2.2. Patients

90 outpatients of the ambulance of the Naturopathic Department with gastrointestinal complaints were included in this observational study. Inclusion criteria were at least 2 of the following symptoms with moderate or greater intensity and frequency during at least 5 of the previous 14 days: bad breath, eructation, nausea, heart burn, stomach discomfort, epigastric pain, diarrhea, hypogastric pain, or spasms. Patients suspected clinically to have organic problems were not included unless procedures for exclusion of organic problems were being performed. Patients who changed medication or diet for their gastrointestinal complaints or with relevance to gastrointestinal functions during the 12 weeks prior to the study or during the study were also not included and not evaluated, respectively. Additionally, patients with diseases that might have influenced symptoms and pregnant or nursing women were not included.

### 2.3. Medication

Silicic acid is an inert adsorption agent that has been used for many years in the treatment of gastrointestinal disorders. It is currently marketed as Silicea Gastrointestinal Gel (manufacturer: Anton Hübner & Co KG) and Sikapur Gastrointestinal Gel (manufacturer: Medopharm GmbH).

At the beginning of treatment, patients were advised to take two 15 mL spoonfuls of gastrointestinal gel dissolved in water or tea 3 times daily at least 2 hours after the last meal or before lunch, dinner, and bedtime, respectively, breakfast. After 3 days, treatment was continued with one 15 mL spoonful three times a day.

### 2.4. Course of Study

Inclusion of patients was based on individual case history and a physical examination. Patients completed questionnaires about gastrointestinal symptoms at the time of inclusion (visit 0), after 3 weeks (visit 2), and after 6 weeks (visit 3). There was also a telephone interview one week (visit 1) after inclusion. Tolerability and compliance were recorded over this 6-week period. Stool behavior and gastrointestinal symptoms were recorded in a patient's diary.

Patients were informed about the test drug, its administration, and how to document consumption, gastrointestinal symptoms, and bowel habits in the diary. They were explicitly instructed to not change their diet during the study. Diet was surveyed using a qualitative questionnaire at the beginning and end of the study. Patients were also weighed at the beginning and end. After 6 weeks, patients were again asked about their condition, medications, and any adverse reactions. Drug tolerability was rated, and physical examinations were repeated. The remaining test drug was weighed as a measure of compliance.

### 2.5. Evaluation Parameters

The primary outcome measure was the change in gastrointestinal symptom score according to a modified Nepean Dyspepsia Index. This score (part I of the validated Nepean Dyspepsia Index [17]) comprised 15 questions related to symptoms of the upper abdomen with points to be summed up from their intensity, frequency, and impact on quality of life with regard to the previous two weeks. We added 10 analogous questions related to symptoms of the lower abdomen. Scores were calculated as the sum of the symptoms for upper abdomen or lower abdomen and total score as the sum of both scores.

### 2.6. Statistical Analysis

All data were evaluated in a descriptive manner. All data are presented as means ± SE. Changes in sum scores were analyzed with two-tailed, paired *t*-tests.

## 3. Results

### 3.1. Patients

90 outpatients were included in the study (64 females, 26 males; mean age 56.5 years (range 19–72); mean body mass index 24.1 (range 17.1–35.0 kg/m^2^)). The most common symptoms were abdominal distension (27 patients), heartburn (21 patients), abdominal cramps (11 patients), and diarrhea (10 patients). Other complaints were nausea (6 patients), epigastric pain (6 patients), and other symptoms (5 patients). 8 patients were smokers, and 34 patients consumed alcohol more than once a week. Physical examination and vital parameters (heart rate and blood pressure) showed no relevant abnormalities. 38 patients received continuous therapy for gastrointestinal symptoms: 12 patients took proton pump inhibitors, 8 took antacids, 4 took acid blockers, 4 took herbal medicines, and 10 others.

67 patients took various other medications (9 patients NSAIDs and 6 thyroid hormones). Change of other nongastrointestinal medications was reported in 1 patient after 1 week, in 8 patients after 3 weeks.

**Figure 1:**
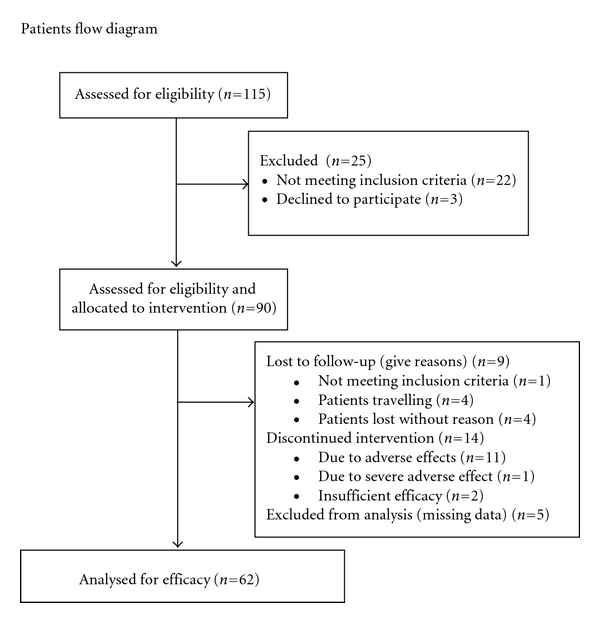
Patients flow chart.

During the course of the study, 23 patients dropped out. 4 of these patients stopped because of travelling, 4 patients dropped out without giving reason, and 1 patient had an exclusion reason which was not documented in V1, 11 patients stopped treatment due to adverse effects (3 of which had a probable relation to the test product), 1 patient due to SUE not related to test product, and 2 patients stopped due to insufficient efficacy. 5 patients did not complete the visit 2 questionnaire. Overall, 62 per-protocol-patients were analyzed for efficacy (see Figure 1).

### 3.2. Outcome

**Figure 2:**
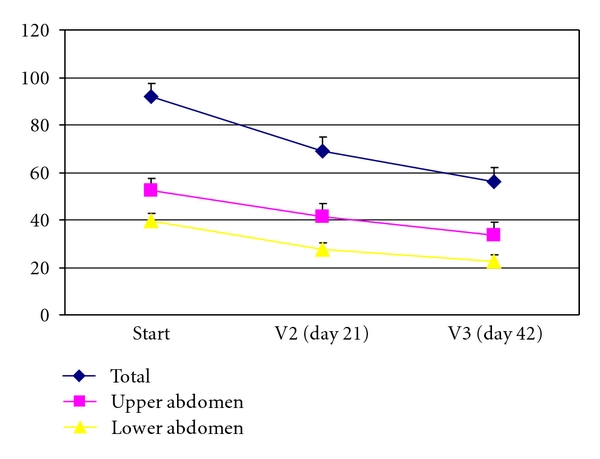
Mean of total symptom score, upper abdomen score, and lower abdomen score in the course of the study: standard error of the mean (*n* = 62).

The mean score for the upper abdomen symptoms decreased after 6-week treatment from 52.2 ± 31.0 to 33.7 ± 28.7 and the mean score for the lower abdomen decreased from 39.6 ± 24.7 to 22.6 ± 21.7. The total gastrointestinal score decreased from 91.8 before therapy to 56.3 after the 6-week treatment (see Figure 2).

Reductions of symptom scores are highly significant as shown in Tables 1 and 2.

Due to the wide range in severity between patients, analysis looked also for relative changes. Mean relative reduction was 30% for upper abdominal symptom score and 42% for lower abdominal symptom score. 37% of the patients were responders (>50% reduction of scores) with regard to upper abdominal complaints, and even 46% with regard to lower abdominal complaints (and 39% for the total gastrointestinal score). 

**Figure 3:**
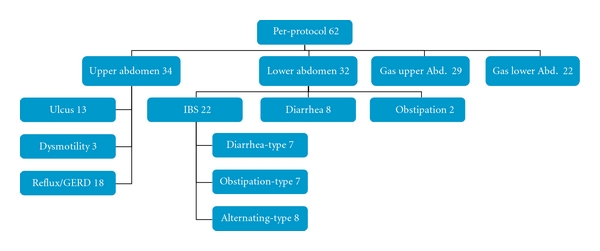
Patients subgroups according their predominant symptoms, patient numbers (*n*).

Patients then were categorized according to their primary complaint at the initial visit as either “upper abdominal” (34 patients) or “lower abdominal” (32 patients). 4 patients had high scores for both categories and were therefore placed in both groups. Other subgroups (with some overlap of patients attributed to several subgroups) are shown in Figure 3.

In addition, patients with severe flatulence were separated nonexclusively into “upper abdominal gas” (29 patients) and “lower abdominal gas” (22 patients) subgroups.

In the upper-abdominal group of symptoms, eructation and upper-abdominal cramps did not improve as much as other symptoms. Improvement of heartburn mainly occurred later than other symptoms between week 3 and 6 of the study; 14 out of 34 (upper abdominal) patients could be characterized as responders for both upper and lower abdominal scores. Patients with ulcer-like symptoms (13 patients) showed a more rapid decrease in symptoms than other groups of patients. However, only 5 of these ulcer-like patients could be considered responders according to total symptom scores. Patients with GERD (18 patients) showed a greater decrease in reflux-related symptoms when compared with other patients—9/18 (50%) patients were responders. 

Out of 23 patients with upper-abdominal gas, 9 were responders. Out of 15 patients with lower-abdominal gas, 6 were responders with parallel relief for flatulence, abdominal cramps, and abnormal bowel habits. 

Out of 22 patients with irritable bowel syndrome, 8 were responders reporting the highest relief for abdominal cramps. Out of 7 patients with constipation-predominant IBS, 4 were responders, mostly due to a decrease in cramping but also due to some improvement with constipation and flatulence. In diarrhea-predominant IBS 2 of 7 were responders, and in IBS-patients with the alternating bowel habit subtype 3 out of 8 patients were categorized as responders due to a improvement in cramps, diarrhea, and gas. 

Patients with diarrhea (without typical IBS-symptoms) had an impressive ratio of 5 responders out of 8. The subgroup lower abdomen with predominant diarrhea complaints improved its symptoms by an average of about 66%. 

### 3.3. Tolerability

Out of the 90 participating patients, 29 reported 35 adverse events and 3 severe adverse events. The latter were exacerbations of known concomitant diseases without causal relation to the gastrointestinal gel but led to 1 dropout. For 11 of the adverse events, a causal relation was rated as possible (3 nausea, 1 abdominal spasm with nausea, constipation, and flatulence, 1 gastritis, 1 abdominal distention, 1 pruritus ani, 1 worsening of heart burn, 1 diarrhea, 1 painful flatulence, 1 gum bleeding) and 5 as probably related (1 pruritus, 1 hypogastric spasm, 1 sleep disorder, 1 nausea, 1 constipation). All of these gastrointestinal complaints disappeared after a few days. 15 adverse events had an improbable relationship to treatment, 7 referring to gastrointestinal symptoms explained by acute viral gastrointestinal infections. Some adverse drug reactions (20 patients) were treated by reduction of dosage (9 patients) or by discontinuing the therapy (11 patients).

## 4. Discussion

This uncontrolled observational study showed a significant and relevant improvement in gastrointestinal symptom scores after 6 weeks of therapy with Silicea Gastrointestinal Gel. Major part of patients reported a marked improvement already after 3 weeks, but improvement continued throughout the second half of the 6-week treatment phase. A detailed questionnaire of gastrointestinal symptoms allowed to define various subgroups and to characterize their symptom reduction. In these subgroup analyses, the effect of symptom reduction could be observed in patients with very different symptom patterns of functional gastrointestinal diseases from nonulcer dyspepsia to irritable bowel syndrome including even constipation-predominant IBS. Mean total scores in all subgroups improved resulting in responder rates over 30%. This is particularly remarkable since most of the patients reported a long history of gastrointestinal complaints resistant to many other therapies. It is also notable that this relief was due to treatment with this rather simple medicinal device silica gel. Good results were observed with a wide variety of symptoms and symptom patterns showing the possible flexibility of treatment with silica gel. 

The good tolerability was documented by adverse event recording during the study. About 6% of patients reported adverse reactions with a probable or possible relationship to the test product. Most of these complaints were transient gastrointestinal symptoms and could also be explained as a kind of complex regulation process within the gastrointestinal tract. 

For functional gastrointestinal symptoms relevant placebo effects are considered. A part of the effect observed in this study may be explained by placebo and regression-to-mean effects. Differences in the improvement of symptoms between subgroups might be a hint that there is no uniform placebo-effect at least. When there would be at least one subgroup with much lower response, this group might serve as a pseudo-control—provided that these differences would not be explained by different placebo-responses according to subgroups. The latter is not well investigated and variation between study settings seems to be of major influence. In our study we could roughly regard the responses in obstipation, in which the mechanism of action of the product is unclear at least as a possible placebo-effect. The responses in other subgroups are better than these in mean. 

Especially in some of the subgroups, small sample sizes are further limiting factors in this pilot study. Further investigation in controlled studies will be necessary to evaluate the effect of Silicea Gastrointestinal Gel on specific functional gastrointestinal syndromes. The presented design of an open study with detailed questionnaires for individual symptoms is a useful tool for testing general gastrointestinal treatments in order to understand which symptoms are most treatable. This pilot study gives direction to future research. 

## 5. Conclusion

Silicea Gastrointestinal Gel might be suitable for uses extending beyond treatment of acute gastrointestinal disorders caused by pathogens. Improvement of various gastrointestinal symptoms in the course of treatment was documented for all patient subgroups, especially in patients suffering from GERD and IBS. Diarrhea without cramps was the single symptom which showed the most significant improvements. 

The maximum effect was observed after several weeks of treatment, with a marked improvement frequently noticeable already after 3 weeks; a further improvement was seen after 6 weeks, and it was observed that there was a progressive reduction of symptoms during treatment until the end of the observation period. 

It is remarkable that responder rates of approximately 30–50% were found in patients who have suffered for years from functional gastrointestinal disorders which might be regarded as therapy-refractory. These patients are highly gratifying for such a simple and well-tolerated medical device. We cannot exclude the results as mainly driven by unspecific placebo-effects. 

Thus the results of this prospective, observational pilot study form a valuable basis for conducting more comprehensive controlled studies in patients with functional gastrointestinal diseases, focusing on diarrhea, GERD, and IBS. 

## Figures and Tables

**Table 1:** Changes of symptom scores for upper abdomen, and lower abdomen (mean, standard deviation, minimum, maximum, *P* value 2sided Wilcoxon-test).

	*N*	Mean	SD	Min	Max	*P* value
V2 (day 21)–V0 upper abdomen	62	10,9	25,0	−40	92	<0.002
V3 (day 42)–V2 (day 21) upper a.	62	7,7	21,2	−51	77	<0.003
V3–V0 upper abdomen	62	18,5	26,6	−26	91	<0.001
V2 (day 21)–V0 lower abdomen	62	12,1	18,0	−32	57	<0.001
V3 (day 42)–V2 (day 21) lower a.	62	4,9	14,0	−32	44	<0.003
V3–V0 lower abdomen	62	17,0	19,5	−27	65	<0.001

**Table 2:** Relative changes between V0 and V3 (mean, standard deviation, minimum, maximum, responder frequency).

	*n*	mean (%)	sd	min	max	responders
Total score	62	38	35.4	− 33	97	24 (39%)
Upper abdomenscore	59^(1)^	30	61.4	−250	100	22 (37%)
Lower abdomen score	61^(1)^	42	42.2	− 62	100	28 (46%)

^(1)^Patients with no complaints for one domain omitted.
